# Lessons Learned in Executing the Nursing Home COVID Action Network

**DOI:** 10.1093/geroni/igab046.1895

**Published:** 2021-12-17

**Authors:** Anna Faul, Samantha Cotton, Pamela Yankeelov, Barbara Gordon

**Affiliations:** 1 University of Louisville, University of Louisville, Kentucky, United States; 2 University of Louisville, Louisville, Kentucky, United States

## Abstract

The University of Louisville ECHO Hub for the Nursing Home COVID Action Network put together a hub of experts that could effectively address the diverse needs of the 240 nursing homes in the 7 cohorts launched. We included an infectious disease expert, a geriatrician, and a behavioral health specialist who adjusted the curriculum to be more in line with the needs of the nursing homes. Our nursing homes were diverse in terms of geography, size and location. We created space for our cohorts to feel comfortable with each other, despite their differences. To foster this sense of togetherness, our facilitators used anonymous opinion polls and incorporated the use of virtual breakout rooms to encourage small group discussions. These strategies assisted in developing a sense of community within the Project ECHO sessions, that will continue to evolve in the post COVID world.

